# Chitinase-3-like protein-1 at hospital admission predicts COVID-19 outcome: a prospective cohort study

**DOI:** 10.1038/s41598-022-11532-x

**Published:** 2022-05-09

**Authors:** Rebecca De Lorenzo, Clara Sciorati, Nicola I. Lorè, Annalisa Capobianco, Cristina Tresoldi, Daniela M. Cirillo, Fabio Ciceri, Patrizia Rovere-Querini, Angelo A. Manfredi

**Affiliations:** 1 Division of Immunology, Transplantation and Infectious Diseases, IRCCS San Raffaele Scientific Institute, San Raffaele Via Olgettina 58, 20132 Milan, Italy; 2 Vita-Salute San Raffaele University, Milan, Italy; 3 Emerging Bacterial Pathogens Unit, IRCCS San Raffaele Scientific Institute, Milan, Italy; 4 Hematology and Bone Marrow Transplant, IRCCS San Raffaele Scientific Institute, Milan, Italy

**Keywords:** Biomarkers, Predictive markers

## Abstract

Infectious and inflammatory stimuli elicit the generation of chitinase-3-like protein-1 (CHI3L1), involved in tissue damage, repair and remodeling. We evaluated whether plasma CHI3L1 at disease onset predicts clinical outcome of patients with Coronavirus 2019 (COVID-19) disease. Blood from 191 prospectively followed COVID-19 patients were collected at hospital admission between March 18th and May 5th, 2020. Plasma from 80 survivors was collected one month post-discharge. Forty age- and sex-matched healthy volunteers served as controls. Primary outcome was transfer to intensive care unit (ICU) or death. CHI3L1 was higher in COVID-19 patients than controls (*p* < 0.0001). Patients with unfavorable outcome (41 patients admitted to ICU, 47 died) had significantly higher CHI3L1 levels than non-ICU survivors (*p* < 0.0001). CHI3L1 levels abated in survivors one month post-discharge, regardless of initial disease severity (*p* < 0.0001), although remaining higher than controls (*p* < 0.05). Cox regression analysis revealed that CHI3L1 levels predict primary outcome independently of age, sex, comorbidities, degree of respiratory insufficiency and systemic inflammation or time from symptom onset to sampling (*p* < 0.0001). Kaplan–Meier curve analysis confirmed that patients with CHI3L1 levels above the median (361 ng/mL) had a poorer prognosis (log rank test, *p* < 0.0001). Plasma CHI3L1 is increased in COVID-19 patients and predicts adverse outcome.

## Introduction

Over one year after the onset of Coronavirus disease 2019 (COVID-19) pandemic, substantial progress has been made in patient management and in understanding disease mechanisms. Several signals have been proposed as putative players in the disease natural history^1–8^. As such, they may be interesting candidates as biomarkers of clinical outcome, helping inform management strategies and minimize the risk of disease progression. The ideal biomarkers should not just reflect the overall inflammatory burden but disclose the events responsible for adverse disease evolution, such as vascular inflammation and lung remodeling^9,10^*.* Moreover, since the interplay of antigen-presenting cells and T cells is crucial determinant of COVID-19 outcome, molecules involved in this process could be suitable candidates^11^. Chitinase-3 like-protein-1 (CHI3L1), a member of the glycoside hydrolase family 18, meets these requirements^12^. CHI3L1 binds to chitin, although being devoid of the ability to cleave the protein. It also binds to other substrates such as hyaluronic acid and heparin. Various signals that are activated in the early phases of COVID-19, including extracellular matrix (ECM) alterations, cell and tissue injury and response to cytokines and growth factors, elicit its synthesis by tissue cells and inflammatory leukocytes^12^. In turn, CHI3L1 stimulates the expression of angiotensin-converting enzyme 2 (ACE-2) and viral spike protein priming proteases in pulmonary epithelial and vascular cells^13^. ACE-2 is the functional receptor of SARS-CoV-2 and mediates viral entry through the viral spike protein^14^. After binding, the spike protein is cleaved by proteases into two subunits that mediate the fusion between viral envelope and cell plasma membrane^14^. ACE is expressed by several cells including enterocytes, renal tubular cells, cardiomyocytes and male reproductive and vascular cells^15^. ACE expression in the respiratory system is limited, in physiological conditions, to regions of the sinonasal cavity and alveolar type II cells^16^. Patients with severe COVID-19 have been recently shown to have higher serum levels of CHI3L1 compared with patients with mild forms^17^. Whether CHI3L1 plasma levels predict clinical outcome remains to be elucidated. The aims of this study were to evaluate plasma CHI3L1 levels in COVID-19 patients at hospital admission and one month after hospital discharge in survivors, and to investigate whether CHI3L1 levels at onset predict adverse outcome.

## Patients and methods

### Patients and study design

This retrospective and prospective investigation includes adult patients (age ≥ 18 years) admitted to San Raffaele University Hospital for COVID-19 between March 18th and May 5th, 2020, and enrolled in the more extensive COVID-BioB observational study^18^. COVID-19 was defined as a positive SARS-CoV-2 real-time reverse-transcriptase polymerase chain reaction (RT-PCR) from a nasopharyngeal swab in the presence of clinical and/or radiologic findings of COVID-19 pneumonia. Blood samples were collected at hospital admission and after viral clearance during scheduled follow-up evaluations in survivors^19–21^ and stored in a dedicated institutional biobank^3^. Detailed demographic, laboratory and clinical data from all patients were recorded in an electronic case record form developed specifically for the study. Forty age- and sex-matched volunteers served as healthy controls (HC). All patients signed an informed consent. The study is compliant with the declaration of Helsinki, was approved by the Hospital Ethics Committee (protocol no. 34/int/2020) and registered on ClinicalTrials.gov (NCT04318366).

### CHI3L1 measurement

Plasma-EDTA samples were obtained by centrifugation of venous blood, immediately frozen and maintained at − 80 °C until subsequent analyses. Plasma was inactivated using tri-(n-butyl) phosphate and Triton X-100 (Sigma) (0.3 and 1%, respectively) for 2 h^22,23^ . CHI3L1 was measured using commercial ELISA kits (Thermo Scientific) following the manufacturer’s instructions.

### Variables and outcome

The following data were considered for all patients: age, sex, comorbidities (i.e. arterial hypertension [HTN], coronary artery disease^2^, diabetes mellitus^24^, chronic obstructive pulmonary disease [COPD], chronic kidney disease [CKD], active neoplasia), laboratory findings at hospital admission (i.e. the ratio of arterial oxygen partial pressure [PaO_2_] in mmHg to fractional inspired oxygen [FiO_2_] expressed as a fraction [PaO_2_/FiO_2_], neutrophil to lymphocyte ratio [NLR], concentration of C-reactive protein [CRP], lactate dehydrogenase^4^, ferritin and D-dimer), time of symptom onset, hospitalization, length of stay, therapy, transfer to the intensive care unit (ICU) and death. CHI3L1 plasma levels at hospital admission and during follow-up in survivors were obtained. A composite outcome including transfer to ICU or death was used as primary outcome.

### Statistical analysis

Categorical variables were expressed as absolute frequencies (percentage) and continuous variables as median (interquartile range [IQR]). Differences in categorical and continuous variables between groups were assessed using Chi-squared or Fisher test, as appropriate, and Mann–Whitney U test, respectively. The Wilcoxon signed‐rank test was used to perform paired comparisons of CHI3L1 levels between hospital admission and follow-up in survivors. Spearman’s correlation test was used to investigate the relationship between CHI3L1 levels and PaO_2_/FiO2, CRP, NLR and LDH. Multivariable Cox regression analysis was performed to investigate the impact of CHI3L1 on the primary outcome when adjusting for age, sex, number of comorbidities, degree of respiratory insufficiency (PaO_2_/FiO_2_), systemic inflammation (CRP levels) and time from symptom onset to blood draw. Variables that showed substantial clinical redundancy with other variables (i.e. CRP *vs.* NLR) were excluded from multivariable analysis to prevent model overfitting. Moreover, multiple multivariable Cox regression analyses were performed to investigate the predictive capacity of CHI3L1 on adverse outcome when adjusting for each variable. Kaplan–Meier analysis and log rank test were used to compare the rates of adverse outcome (transfer to ICU or death) or mortality in patients with CHI3L1 levels below or above the median. Statistical analyses were performed using R statistical package (version 4.0.0, R Foundation for Statistical Computing, Vienna, Austria), with a two-sided significance level set at *p* < 0.05.

## Results

### Patient characteristics

One hundred ninety-one COVID-19 patients admitted to a tertiary-care hospital during the first wave of the pandemic were included in the study. Demographical, clinical and laboratory characteristics are reported in Table 1. Median (IQR) time from hospital admission to blood draw was 1 (0–1) days. At time of sampling, 150 (79%) patients had not received any treatment. Twenty-five (13%) patients had started hydroxychloroquine, 10 (5%) steroids, 7 (4%) lopinavir/ritonavir, 23 (12%) low molecular weight heparin (LMWH) for a median time of 1 (0–1) days. Most patients were males (63.9%) and median (IQR) age was 61.8 (50.1–72.3) years. The most frequent comorbidity was HTN (41.1%), followed by DM (20.4%) and CAD (11.5%). The majority of patients were hospitalized (80.1%) for a median (IQR) time of 12 (4–24) days. Steroid therapy was administered to 23% of patients and 45.5% of patients received LMWH. Forty-one (21.5%) patients were transferred to the ICU and 47 (24.6%) died, after a median (IQR) time of 5 (1–8) and 12 (5–21) days, respectively.

**Table 1:** Characteristics of COVID-19 patients.

	Overall	ICU/death		*P* value
	n = 191	No n = 125	Yes n = 66	
Age (years)	61.8 (50.1–72.3)	57.7 (48.4–67.4)	66.2 (57.7–77.3)	0.0003
Female sex	69 (36.1)	51 (40.8)	18 (27.3)	0.09
**Comorbidities**				
HTN	79 (41.4)	45 (36)	34 (51.5)	0.055
COPD	10 (5.2)	4 (3.2)	6 (9.1)	0.16
CAD	22 (11.5)	8 (6.4)	14 (21.2)	0.005
DM	39 (20.4)	19 (15.2)	20 (30.3)	0.02
Active neoplasia	6 (3.1)	3 (2.4)	3 (4.5)	0.7
CKD	17 (8.9)	6 (4.8)	11 (16.7)	0.01
Time from symptom onset to blood draw (days)	8 (4–11)	8 (5–11)	6.5 (3–9)	0.003
**At hospital admission**				
PaO_2_/FiO_2_	276.2 (190.5–333.8)	309.5 (255.4–361.9)	159.1 (73.8–266.7)	< 0.0001
NLR	5.3 (3.5–8.5)	4.4 (2.9–6.7)	9.2 (5.4–13.2)	< 0.0001
CRP (mg/dL)	79.2 (30.6–152.6)	60.1 (17.6–116.8)	156.8 (82.8–231.4)	< 0.0001
Hospitalization	153 (80.1)	88 (70.4)	65 (98.5)	–
Length of stay (days)		10 (2–19)	19 (7–35)	0.0001
Steroid therapy	44 (23)	15 (12)	29 (43.9)	< 0.0001
LMWH therapy	87 (45.5)	48 (38.4)	39 (59.1)	0.0004
ICU transfer	41 (21.5)	–	41 (62.1)	–
Death	47 (24.6)	–	47 (71.2)	–

Categorical variables were expressed as count (percentage), while continuous variables as median (interquartile range).

*ICU* Intensive care unit, *HTN* Arterial hypertension; *COPD* Chronic obstructive pulmonary disease, *CAD* Coronary artery disease, *DM* Diabetes mellitus, *CKD* Chronic kidney disease; *PaO*_*2*_*/FiO*_*2*_ Ratio of arterial oxygen partial pressure to fractional inspired oxygen, *NLR* Neutrophil to lymphocyte ratio, *CRP* C-reactive protein, *LMWH* Low-molecular weight heparin.

### Early and transient increase of CHI3L1 plasma levels in COVID-19

**Figure 1:**
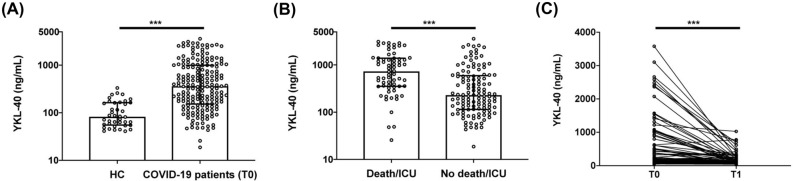
CHI3L1 plasma levels in COVID-19 patients and healthy controls. CHI3L1 plasma levels in: (**A**) age- and sex-matched healthy controls (HC) and COVID-19 patients at hospital admission (COVID-19 patients T0); (**B**) COVID-19 patients transferred to ICU or who died (Death/ICU) and patients with favorable outcome (No death/ICU); (**C**) eighty survivors at hospital admission (T0) and one month after discharge (T1). *** < 0.0001.

**Figure 2:**
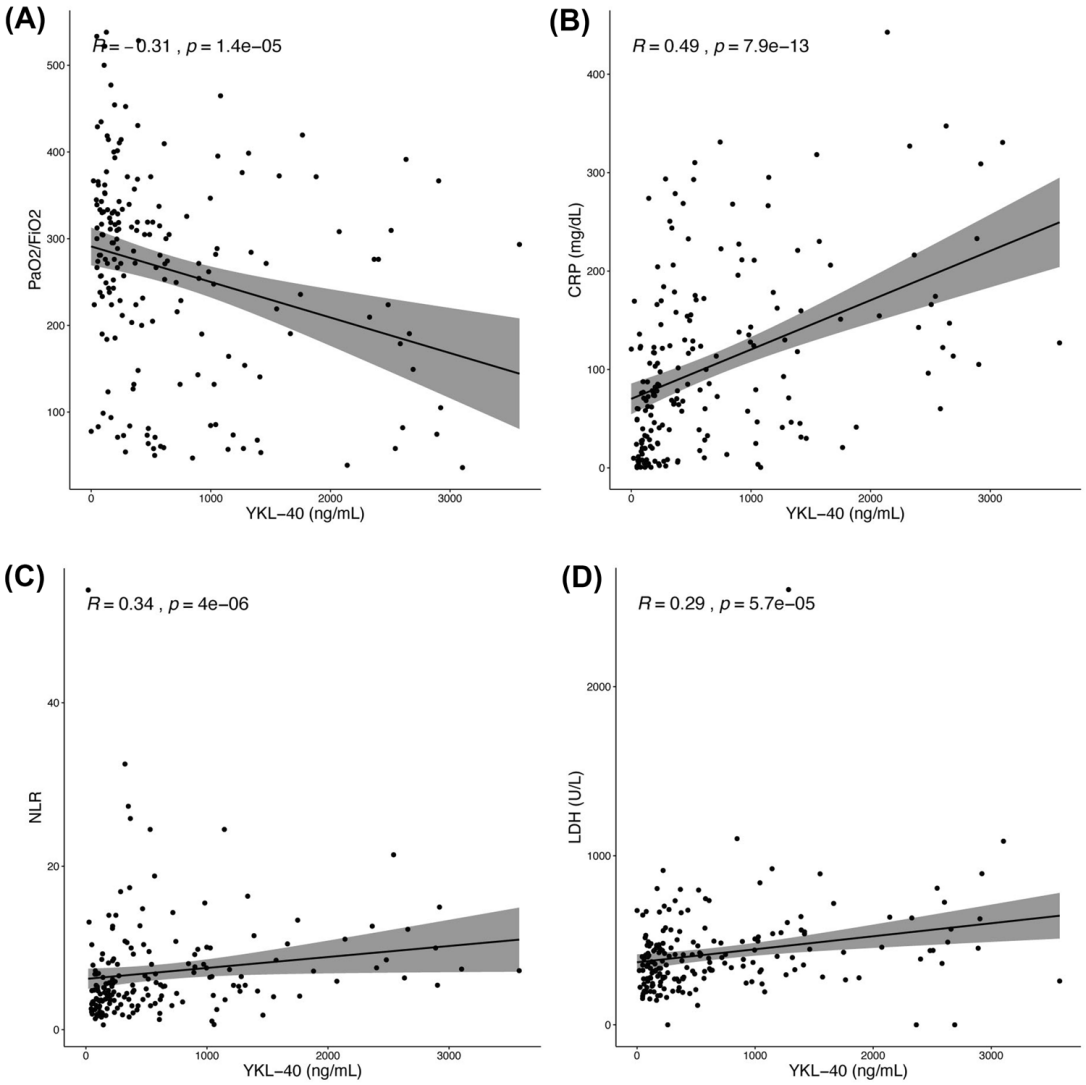
Correlations of CHI3L1 plasma levels with PaO_*2*_/FiO2, CRP, NLR and LDH levels. Spearman’s test was used to correlate CHI3L1 levels with PaO_2_/FiO2 (**A**), CRP (**B**), NLR (**C**) and LDH (**D**) values at the hospital admission.

CHI3L1 plasma levels were significantly higher in COVID-19 patients at hospital admission (T0) than in HC (361.0 [152.9–988.8] ng/mL vs 82.4 [56.1–162.0] ng/mL, *p* < 0.0001, Fig. 1 panel A). Patients transferred to ICU or who died had higher plasma levels of CHI3L1 at admission than those with a favorable outcome (730.4 [354.7–1375.4] ng/mL vs 229.1 [115.3–579.9] ng/mL, *p* < 0.0001, Fig. 1 panel B). Specifically, the CHI3L1 plasma level was 260 [129.2–822.7] ng/mL in survivors and 533.54 [344.5–1309.7] ng/mL in patients who died (*p* = 0.0008). Similarly, CHI3L1 level was 1027.2 [421.8–1665] ng/mL in patients transferred to ICU and 259.63 [130.7–610.3] ng/ml in those who needed less intensive care (*p* < 0.0001). Eighty patients who survived the acute phase of the disease underwent a second blood draw after a median (IQR) time from hospital discharge of 40 (30–52) days. At paired analysis, CHI3L1 plasma levels of COVID-19 survivors after hospital discharge (T1) were significantly lower compared with levels at admission (T0) (232.1 [126.0–942.0] ng/mL vs 118.5 [70.2–236.7] ng/mL, *p* < 0.0001, Fig. 1 panel C), although remaining higher than HC (*p* < 0.05). CHI3L1 levels at admission were inversely correlated with respiratory function, as reflected by the PaO_2_/FiO_2_ (R coefficient − 0.31, *p* < 0.0001, Fig. 2 panel A). On the contrary, CHI3L1 levels positively correlate with CRP levels (R coefficient 0.49, *p* < 0.0001, Fig. 2 panel B), NLR (R coefficient 0.34, *p* < 0.0001, Fig. 2 panel C) and LDH levels (R coefficient 0.29, *p* < 0.0001, Fig. 2 panel D).

### CHI3L1 levels independently predict COVID-19 clinical outcomes

**Figure 3:**
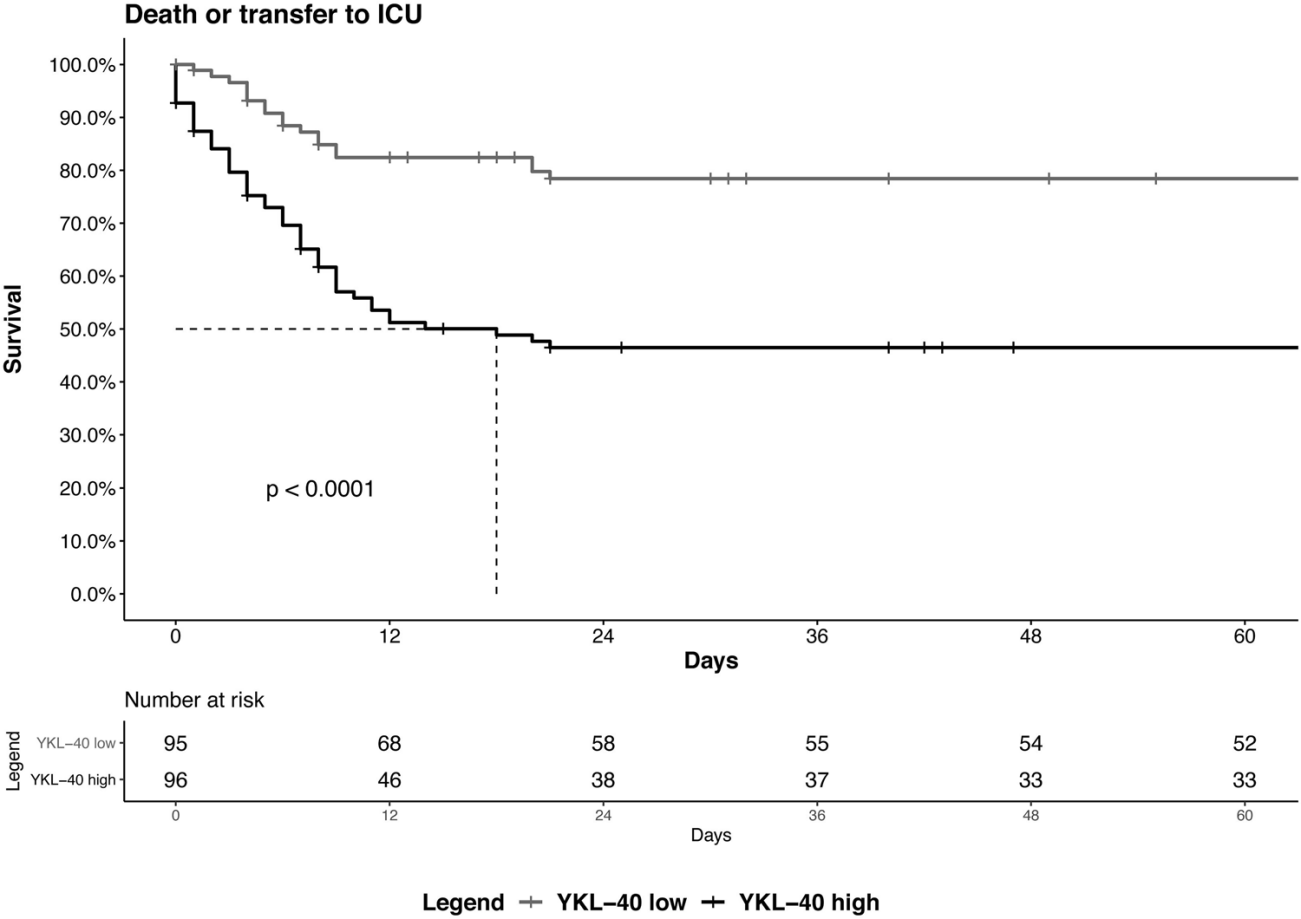
Kaplan–Meier curves depicting rates of adverse autcome (transfer to ICU or death) in patients with CHI3L1 levels below (low) or above (high) the median value of 361 ng/mL. Log rank test, *p* < 0.0001.

Multivariable Cox regression analysis revealed that CHI3L1 plasma levels (ng/mL) at admission predict the risk of adverse outcome (transfer to ICU/death) independently of age, sex, comorbidities, degree of respiratory insufficiency and systemic inflammation (CRP) at admission, and time from symptom onset to blood draw (*p* < 0.01, Table 2). Risk of adverse outcomes was increased by 0.5% per increase of 10 ng/mL of CHI3L1 plasma levels. Kaplan–Meier curve analyses confirmed that patients with plasma levels of CHI3L1 above the median value of 361 ng/mL had a significantly higher risk of adverse outcome (transfer to ICU or death, log rank test, *p* < 0.0001, Fig. 3) and of death (log rank test, p 0.0036, Supplementary Fig. 1) than those with lower levels. Multivariable Cox regression analyses were performed to investigate whether the ability of CHI3L1 to predict adverse outcome was independent of patient characteristic. CHI3L1 levels (ng/mL) predict transfer to ICU or death independent of HTN, COPD, CAD, DM, active neoplasia, CKD, NLR at admission, hospitalization, length of stay in hospital, steroid therapy and LMWH administration during hospitalization (Supplementary Table 1).

**Table 2:** Multivariable Cox regression analysis predicting transfer to ICU/death.

	HR	95% CI	*P* value
CHI3L1 (for every increase of 10 ng/mL)	1.005	1.001–1.008	0.005
Age (years)	0.983	0.960–1.006	0.148
Female sex	0.578	0.323–1.034	0.065
Number of comorbidities	1.471	1.151–1.880	0.002
PaO_2_/FiO_2_	0.993	0.990–0.996	0.000
CRP (mg/dL)	1.001	0.998–1.004	0.671
Time from symptom onset to blood draw (days)	0.968	0.910–1.031	0.316

*HR* Hazard ratio, *95% CI* 95% confidence interval, PaO_2_/FiO_2_ Ratio of arterial oxygen partial pressure to fractional inspired oxygen, *CRP* C-reactive protein.

## Discussion

We report that CHI3L1 accumulates early in COVID-19 patients. Patients with higher CHI3L1 levels had higher CRP levels and NLR values, reflecting a high inflammatory burden. These results agree well with those recently reported by^17^, who retrospectively compared serum levels of CHI3L1 in hospitalized COVID-19 patients, healthy subjects, patients with chronic obstructive pulmonary disease and with unrelated interstitial lung disease.

We found that plasma levels of CHI3L1 were reduced in COVID-19 survivors four weeks after clinical remission, at a time when systemic inflammation had returned to baseline levels^25^. Interestingly, CHI3L1 levels at this point were still significantly higher than in healthy subjects, suggesting that stimuli sustaining CHI3L1 production are still present. The crosstalk between the ECM and CHI3L1^12^ is intriguing since ECM remodeling occurs during acute COVID-19^26^, and may persist following disease resolution given the evidence of autoantibodies against ECM components in COVID-19 patients^27^.

High levels of CHI3L1 were associated with an increased risk of adverse outcome, including transfer to the ICU or mortality, independently of age, sex, comorbidities, degree of respiratory insufficiency and systemic inflammation at admission, and time from symptom onset to blood draw, all known to be associated with COVID-19 clinical outcome^28,29^. This observation indicates that the early generation of CHI3L1 is integral to the response initiated by the host recognition of SARS-CoV-2 and does not only reflect the magnitude of the inflammatory response or the clinical status. CHI3L1 is a recognized biomarker of kidney injury^30,31^, which makes it tempting to speculate that CHI3L1 levels may at least in part reflect kidney damage^32^. To verify this possibility, data on larger groups of patients are needed. Further studies are warranted to identify the stimulus involved in the persistent generation of CHI3L1 in patients with COVID-19 and the reasons of its preferential early accumulation in patients who will experience adverse outcomes. This might prove valuable to dissect at the molecular level the heterogeneity of the disease, possibly allowing a better understanding of the mechanisms responsible for COVID-19 complications.

Our study has limitations. First, the monocentric nature of the cohort implies that conclusions need to be further validated in larger independent cohorts. Second, although patients were prospectively followed, blood specimens at follow-up were available for only eighty of them. Third, a longer follow-up might add information, especially after stratifying survivors based on long-term *sequelae*^19,33^. In addition, the limited sample size may hamper the generalizability of results. Validation of our findings in larger cohorts support the routine use of CHI3L1 for prognostic and patient management purposes. Although quantification of CHI3L1 plasma levels is easy to perform through ELISA technique, it remains more expensive and time-consuming than measuring conventional biomarkers such as CRP, LDH or NLR, whose specificity for COVID-19 is however limited. Altogether, our data indicate that plasma levels of CHI3L1, a major player in the host response to inflammatory threats, are increased in patients with more severe COVID-19 patients and predicts adverse outcome independently of systemic inflammation and clinical indicators of severity. CHI3L1 may play a role in the progression of COVID-19 through mechanisms not directly related to the extent of the host inflammatory response. Further studies are needed to verify this claim.

## Supplementary Information

Supplementary Information 1.

Supplementary Information 2.

Supplementary Information 3.

## Data Availability

The data underlying this article will be shared on reasonable request to the corresponding author.
