# Supplementary material for: Chitinase-3-like protein-1 at hospital admission predicts COVID-19 outcome: a prospective cohort study
Source: Sci Rep. 2022 May 9;12:7606. doi: 10.1038/s41598-022-11532-x (PMC9084263; doi:10.1038/s41598-022-11532-x)
Supplement: Supplementary file 1 — Supplementary Information 1. [file 41598_2022_11532_MOESM1_ESM.docx]

|  | Patient characteristics | | |  | CHI3L1 (ng/mL) | | |
| --- | --- | --- | --- | --- | --- | --- | --- |
|  | Variables | HR (95% CI) | P value |  | HR (95% CI) | P value | |
|  | *Comorbidities* |  |  |  |  |  | |
|  | HTN | 1.54 (2.5-0.95) | 0.019 |  | 1.006 (1.003-1.008) | <0.0001 | |
|  | COPD | 3.004 (1.27-7.09) | 0.012 |  | 1.006 (1.0043-1.008) | <0.0001 | |
|  | CAD | 2.28 (1.26-4.13) | 0.006 |  | 1.005 (1.003-1.008) | <0.0001 | |
|  | DM | 1.65 (0.97-2.8) | 0.064 |  | 1.005 (1.004-1.008) | <0.0001 | |
|  | Active neoplasia | 1.6 (0.5-5.15) | 0.43 |  | 1.006 (1.003-1.008) | <0.0001 | |
|  | CKD | 2.59 (1.35-4.96) | 0.004 |  | 1.006 (1.003-1.008) | <0.0001 | |
|  | *At hospital admission* |  |  |  |  |  | |
|  | NLR | 1.049 (1.02-1.07) | <0.0001 |  | 1.005 (1.002-1.008) | <0.0001 | |
|  | Hospitalization | 11.67 (1.61-84.49) | 0.015 |  | 1.005 (1.002-1.007) | <0.0001 | |
|  | Length of stay (days) | 1.01 (1.004-1.02) | 0.004 |  | 1.005 (1.003-1.008) | <0.0001 | |
|  | Steroid therapy | 3.21 (1.87-5.52) | <0.0001 |  | 1.005 (1.003-1.008) | <0.0001 | |
|  | LMWH therapy | 1.83 (1.03-3.27) | 0.04 |  | 1.005 (1.003-1.008) | <0.0001 | |
| Each row reports the hazard ratio, HR, (95% confidence interval, CI) and p value of each variable and CHI3L1 when they are used as covariates at multivariable Cox regression analysis. Abbreviations: ICU, intensive care unit; CHI3L1, Chitinase-3-like protein-1, HTN, arterial hypertension; COPD, chronic obstructive pulmonary disease; CAD, coronary artery disease; DM, diabetes mellitus; CKD, chronic kidney disease; NLR, neutrophil to lymphocyte ratio; LMWH, low-molecular weight heparin. | | | | | | |  |

Supplementary table 1. Multivariable Cox regression analyses predicting ICU/death in COVID-19 patients.
