# Supplementary figures and images for: Chitinase-3-like protein-1 at hospital admission predicts COVID-19 outcome: a prospective cohort study
Source: Sci Rep. 2022 May 9;12:7606. doi: 10.1038/s41598-022-11532-x (PMC9084263; doi:10.1038/s41598-022-11532-x)

# Death

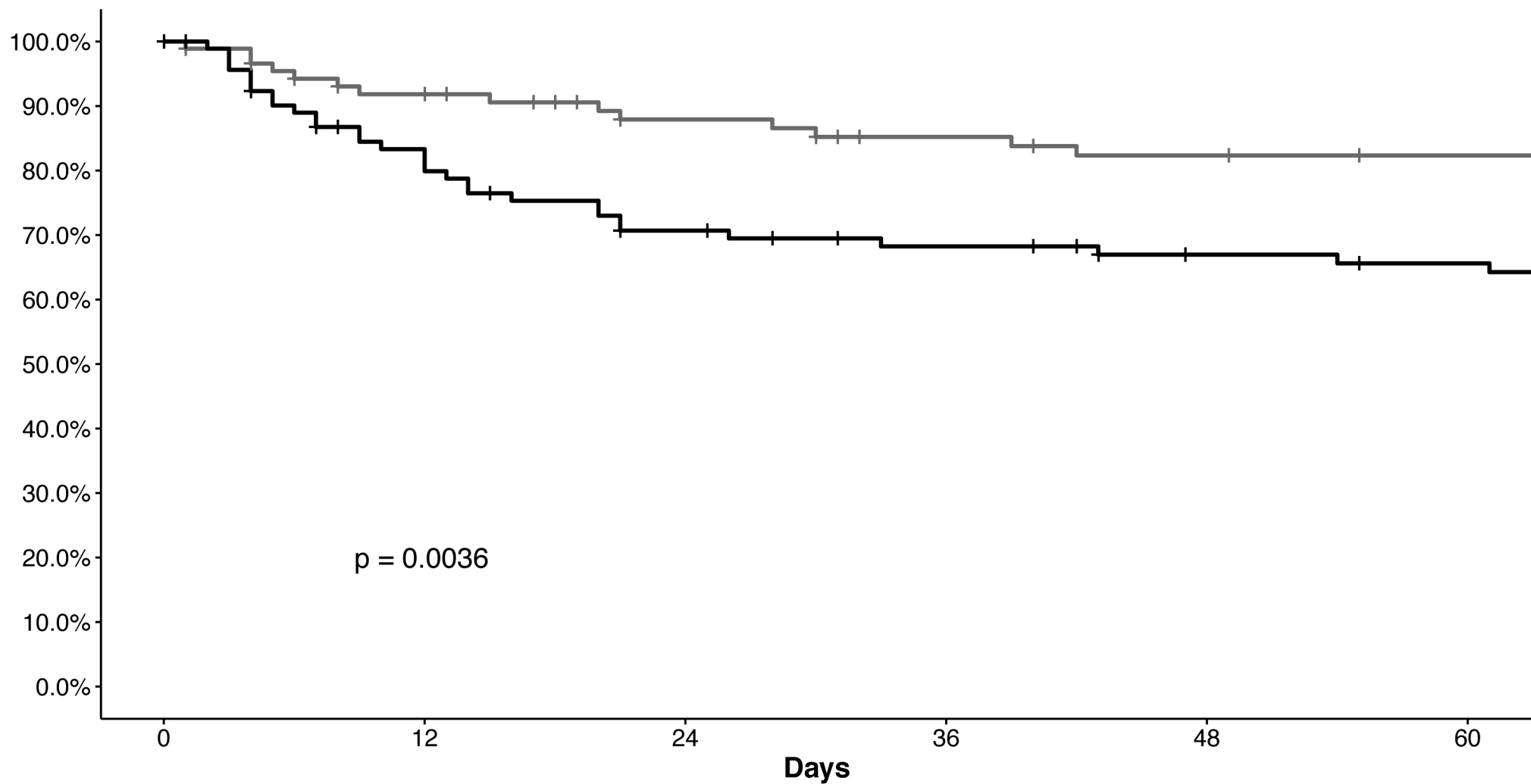

## Number at risk

|             |    |    |    |    |    |    |
|-------------|----|----|----|----|----|----|
| YKL-40 low  | 95 | 76 | 65 | 60 | 57 | 55 |
| YKL-40 high | 96 | 73 | 60 | 55 | 50 | 48 |

Legend + YKL-40 low + YKL-40 high

Supplement: Supplementary file 2 — Supplementary Information 2. [file 41598_2022_11532_MOESM2_ESM.pdf]
