# Supplementary material for: Chitinase-3-like protein-1 at hospital admission predicts COVID-19 outcome: a prospective cohort study
Source: Sci Rep. 2022 May 9;12:7606. doi: 10.1038/s41598-022-11532-x (PMC9084263; doi:10.1038/s41598-022-11532-x)
Supplement: Supplementary file 3 — Supplementary Information 3. [file 41598_2022_11532_MOESM3_ESM.docx]

Supplementary Figure 1. Kaplan-Meier curves depicting mortality rates in patients with CHI3L1 levels below (low) or above (high) the median value of 361 ng/mL. Log rank test, p 0.0036.
